# Endovenous laser ablation versus radiofrequency ablation in chronic venous insufficiency: a comparative meta-analysis of efficacy and safety

**DOI:** 10.3389/fmed.2026.1816752

**Published:** 2026-08-27

**Authors:** Shuai Li, Weilin Wang, Lan Zhang

**Affiliations:** Department of Vascular Surgery, Taizhou Hospital of Zhejiang Province Affiliated to Wenzhou Medical University, Taizhou, Zhejiang, China

**Keywords:** chronic venous insufficiency, endovenous laser ablation, meta-analysis, radiofrequency ablation, systematic review

## Abstract

**Background:**

Chronic venous insufficiency (CVI) is a common vascular disorder that substantially impairs quality of life. Endovenous laser ablation (EVLA) and radiofrequency ablation (RFA) have largely replaced conventional surgery; however, their comparative efficacy and safety—particularly across follow-up time points and EVLA wavelengths—remain debated.

**Methods:**

We conducted a systematic review and meta-analysis of PubMed, Scopus, Web of Science, the Cochrane Library, and Google Scholar from inception to September 2024. Thirty-three comparative studies (9 randomized controlled trials and 24 non-randomized studies) involving 22,814 patients (8,144 EVLA and 14,670 RFA) were included. Analyses were performed at the patient level. The single primary outcome was complete occlusion of the treated vein; all other endpoints were secondary. Secondary outcomes included procedural success, recanalization, partial occlusion, reflux-free rate, postoperative pain, recurrence, return to work or daily activities, patient satisfaction, and complications, with stratified analyses by follow-up time and EVLA wavelength.

**Results:**

RFA was associated with significantly lower postoperative pain at 6 weeks (mean difference [MD] = −3.00; 95% CI: −3.42 to −2.58) and 6 months (MD = −1.00; 95% CI: −1.85 to −0.14), and a reduced risk of paresthesia at 1 month (odds ratio [OR] = 0.52; 95% CI: 0.28–0.95). Non-randomized studies suggested lower recurrence rates with RFA compared with EVLA (OR = 0.42; 95% CI: 0.29–0.62), although randomized trials showed no significant difference. Complete occlusion rates were lower with RFA than with EVLA in pooled analyses (OR = 0.07; 95% CI: 0.04–0.14), particularly at early follow-up (1 day: OR = 0.01; 95% CI: 0.00–0.11), with differences diminishing over time. Variation in outcome definitions and imaging timing likely contributed to these early effects. Reflux-free rates and patient-reported outcomes, including Aberdeen Varicose Vein Questionnaire scores, did not differ significantly between groups.

**Conclusion:**

Both EVLA and RFA are effective and safe treatments for CVI. RFA provides advantages in short-term postoperative comfort and selected complications, whereas EVLA—particularly with 1,470-nm wavelengths—achieves more consistent early technical occlusion. However, these differences do not translate into clear differences in reflux-free status or patient-reported quality-of-life outcomes, supporting individualized treatment selection. When performed competently, neither modality offers a clinically meaningful advantage for patient-centered outcomes.

## 1 Introduction

Chronic venous insufficiency (CVI) is a prevalent and debilitating condition affecting up to 40% of the population, with a higher incidence in women and older individuals (1). Characterized by venous reflux, venous hypertension, and progressive venous damage, CVI often presents as varicose veins, edema, skin changes, and, in severe cases, venous ulcers (2). Left untreated, CVI can significantly impair quality of life, leading to chronic pain, mobility limitations, and a higher risk of complications such as venous thromboembolism (3). Therefore, effective management is critical to alleviate symptoms, prevent progression, and improve patient outcomes.

For decades, conventional surgery, particularly high ligation and stripping of the saphenous vein, was the gold standard for treating CVI. However, these surgical methods are associated with significant postoperative pain, prolonged recovery times, and recurrence (4). In response to these challenges, minimally invasive endovenous therapies, including endovenous laser ablation (EVLA) and radiofrequency ablation (RFA), have emerged as preferred alternatives, offering shorter recovery times and improved patient satisfaction (5). Both procedures achieve vein closure by delivering thermal energy within the vein, causing endothelial damage, vein wall collapse, and eventual fibrosis (6).

While RFA and EVLA are now widely used, it remains unclear which technique provides superior long-term outcomes, as both have demonstrated high success rates and similar safety profiles in previous studies (7–11). However, differences in postoperative recovery, occlusion rates, recurrence, and complications such as pain and paresthesia warrant further investigation. Additionally, the performance of EVLA may vary depending on the laser wavelength used, with longer wavelengths (e.g., 1,470-nm) now routinely used in clinical practice due to their more controlled and effective ablation properties compared to earlier wavelengths like 810-nm and 980-nm (8, 10, 12, 13). Given these nuances, a thorough comparison of these two treatment modalities is essential to guide clinical decision-making.

This systematic review and meta-analysis aim to provide a comprehensive comparison of EVLA and RFA in patients with CVI, focusing on key clinical outcomes. By synthesizing data from both randomized controlled trials and non-randomized studies, we seek to clarify the relative advantages of each technique and offer insights that can inform personalized treatment strategies in the management of CVI.

## 2 Materials and methods

### 2.1 Design and literature search

This systematic review and meta-analytic study included original research that compared RFA to EVLA in patients with CVI. This work has been reported in line with the PRISMA (14) (Preferred Reporting Items for Systematic Reviews and Meta-Analyses) Guidelines. This review was not prospectively registered in PROSPERO due to the presence of an existing registered protocol addressing a similar research question (PROSPERO ID: CRD42025629845), in line with current PROSPERO guidance to avoid duplication.

We searched PubMed, Scopus, Web of Science, Cochrane Library, and Google Scholar (first 200 citations) (15) up to September 2nd, 2024. The search combined terms related to chronic venous insufficiency, endovenous ablation, laser therapy, and radiofrequency ablation. No restrictions were applied regarding the original language of publication. The detailed search strategy is provided in Supplementary Table S1.

### 2.2 Selection strategy

Studies were selected using the PICOS (Population, Intervention, Comparison, Outcomes, Study Design) framework (16). The inclusion criteria included:

- Population: patients with CVI who did not undergo prior EVLA or RFA
- Intervention: RFA
- Comparison: EVLA of any wavelength
- Outcomes: any efficacy or safety endpoint (a full list is provided below)
- Study design: both randomized controlled trials (RCTs) and non-randomized studies of intervention (NRSI) either cohort or case control studies

The exclusion criteria included the following:

- Patients with varicose veins of unknown etiology
- Studies reporting other treatment modalities (i.e., microwave ablation)
- Non-comparative studies
- Lack of outcome data
- Non-original research (i.e., reviews, commentaries, editorials, etc.)
- Case reports and case series
- Overlapping datasets or duplicated studies
- Study protocols

All retrieved records were imported into EndNote (Clarivate Analytics, version X8), where duplicate citations were identified and removed using the software’s automated duplicate detection function authors-, title-, and year of publication-based, followed by manual verification to ensure accurate de-duplication prior to title and abstract screening.

### 2.3 Data collection and outcomes

The senior author designed the data collection sheet using Microsoft Excel. The first part covered studies’ (design, country of investigation, follow-up), patients’ characteristics (sample, age, gender, operated side, bilaterality, presentation with varicose veins, involved veins, adjunct treatments, anesthesia type, veins’ diameter, length of treated vein segment, and severity at baseline), and intervention-related data (EVLA wavelength, type of RFA). The severity of CVI at baseline was reported using CEAP (Clinical, Etiological, Anatomical, and Pathophysiological) classification, where CEAP 4–6 indicated severe disease (17). CVI severity was also assessed using the Venous Clinical Severity Score (VCSS) at baseline.

The second part covered our outcomes. The primary outcome was complete occlusion of the treated vein, the principal objective measure of technical efficacy and the single prespecified primary endpoint; all other efficacy and safety measures were prespecified as secondary. Secondary efficacy outcomes included procedural success, recanalization, partial occlusion, and reflux-free rates. Success was defined based on duplex ultrasound findings and included: complete occlusion of the treated vein with partial recanalization of a treated vein < 5 mm in length (8), lack of blood flow in the treated vein segment <3 cm (18), and absence of reflux throughout the treated vein segment (19). Given the limited sample, a subgroup analysis based on definition criteria was not feasible. The definition criteria of measured outcomes are provided in Supplementary Table S2.

However, given that these technical outcomes do not always directly translate into patient-reported benefits, we also examined secondary outcomes, which are more patient-centered, including postoperative pain using the Visual Analogue Scale (VAS), VCSS score, Aberdeen Varicose Vein Questionnaire (AVVQ), satisfaction, return to work and daily activities, recurrence, and complications. These secondary outcomes are arguably more relevant to real-world clinical decision-making.

### 2.4 Risk of bias assessment

The methodological quality of RCTs was assessed using the revised Cochrane Risk of Bias tool (RoB-II). This tool evaluates five domains: (1) bias arising from the randomization process, (2) bias due to deviations from intended interventions, (3) bias due to missing outcome data, (4) bias in measurement of the outcome, and (5) bias in selection of the reported result. Each domain was judged as “low risk,” “some concerns,” or “high risk” according to the RoB-II signaling questions and algorithm. An overall judgment of “low risk of bias” was assigned if all domains were rated as low risk; “some concerns” if at least one domain raised some concerns but none were rated as high risk; and “high risk of bias” if at least one domain was rated as high risk or if multiple domains raised some concerns (20). The quality of NRSIs was assessed using the Newcastle-Ottawa Scale (NOS), which evaluates studies across three domains: selection (maximum of 4 stars), comparability (maximum of 2 stars), and outcome/exposure assessment (maximum of 3 stars). Studies were classified as good quality if they received 3–4 stars in selection, 1–2 stars in comparability, and 2–3 stars in outcome/exposure; fair quality if they received 2 stars in selection, 1–2 stars in comparability, and 2–3 stars in outcome/exposure; and poor quality if they received 0–1 star in selection, 0 stars in comparability, or 0–1 star in outcome/exposure.

### 2.5 Statistical analysis

All analyses were conducted using STATA v18 (StataCorp, USA), with RFA as the intervention and EVLA as the control. Per protocol, outcomes with fewer than three studies were excluded from meta-analysis (e.g., ecchymosis, tenderness, CIVIQ-20, hospitalization time, GSV diameters, QoL domains, and lab values). When continuous outcomes were reported as medians with IQRs or CIs, they were converted to means and SDs using validated formulas (21, 22).

Due to clinical heterogeneity—variability in EVLA wavelengths and follow-up durations—pooled meta-analysis was not feasible. Instead, subgroup analyses were performed by study design (RCT vs. NRSI), follow-up time, and EVLA wavelength. All meta-analyses used the random-effects model via the REML method (metan command) (23), with heterogeneity assessed using the *I*^2^ statistic (*I*^2^ > 40% and *p* < 0.05 considered significant) (24). Sensitivity analyses included Galbraith plots to detect outliers, and publication bias was assessed using funnel plots and asymmetry tests when ≥10 studies were available (25).

Meta-regression explored determinants of treatment effect. While several covariates were considered (e.g., age, adjunct treatments, disease severity, GSV diameter), only study design, follow-up time, and EVLA wavelength met the threshold for inclusion due to reporting limitations. Multicollinearity was assessed using variance inflation factors (>5 indicating concern) (26), and model fit was evaluated by adjusted *R*^2^. Only variables reported by ≥5 studies with significant heterogeneity were eligible for subgroup or meta-regression analysis (27).

## 3 Results

### 3.1 Literature search results

**Figure 1:**
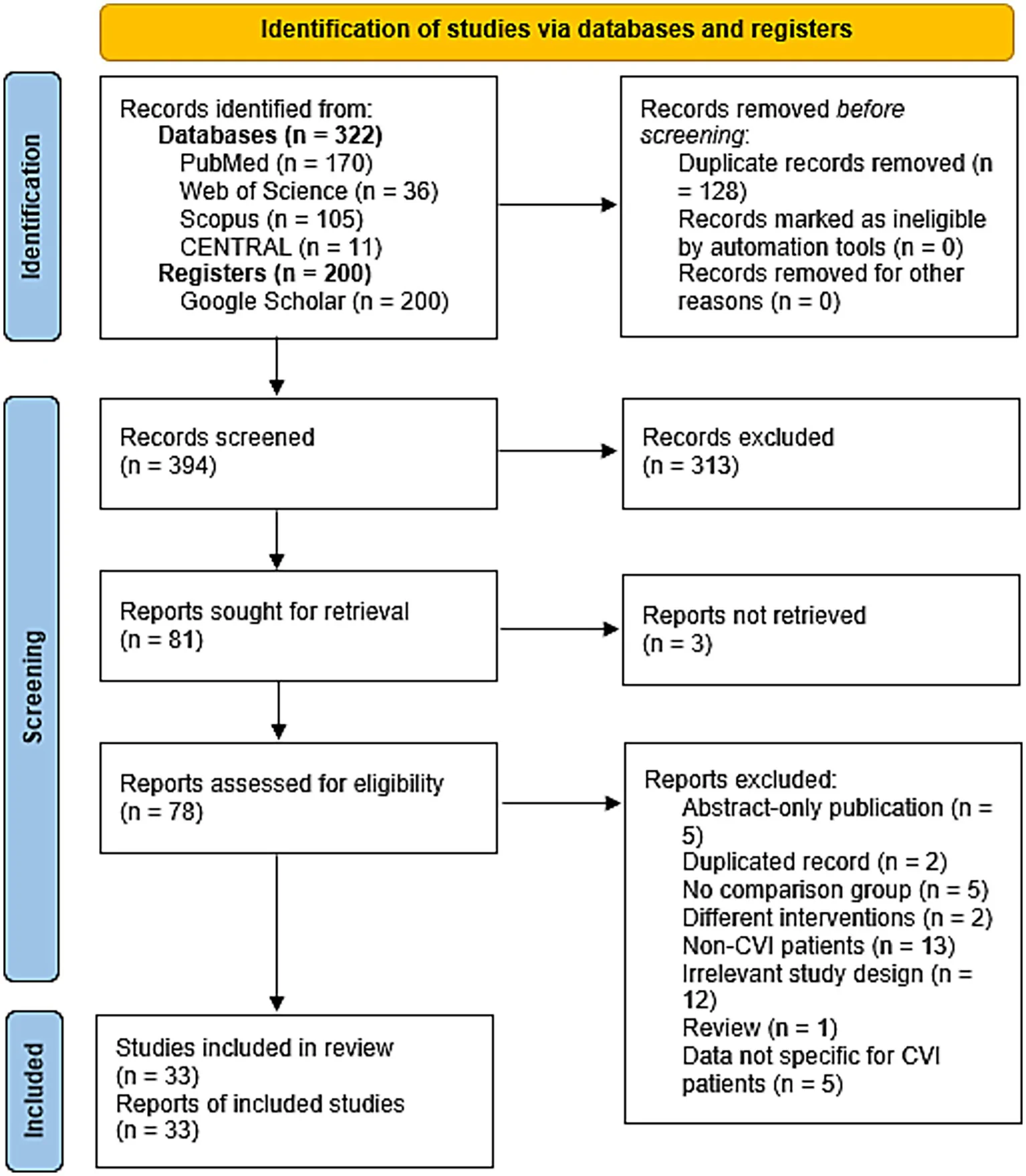
PRISMA 2020 flow diagram of the study selection process, showing the number of records identified through database searching, records remaining after duplicate removal, reports screened at title/abstract and full-text levels, full-text reports excluded with reasons, and the studies finally included in the systematic review and meta-analysis.

The literature search and screening process yielded 522 citations, with 128 duplicates identified using EndNote (Figure 1). After de-duplication, 394 records remained; 313 were excluded during title and abstract screening. Of the 81 full-texts sought, 78 were retrievable and assessed for eligibility. Forty-five studies were excluded due to abstract-only format (*n* = 5), duplicates (*n* = 2), no comparison group (*n* = 5), unrelated interventions (*n* = 2), non-CVI populations (*n* = 13), ineligible design (*n* = 12), review articles (*n* = 1), and data not specific to CVI patients (*n* = 5). One study involving repeat procedures was excluded (28), and another lacked group sizes despite providing outcome data for both procedures; the authors did not respond to inquiries within 30 days, leading to exclusion 7. Ultimately, 33 studies were included in the final synthesis (7–13, 18, 19, 29–52).

### 3.2 Baseline characteristics of included studies

Table 1 summarizes the included studies, comprising 9 randomized trials and 24 non-randomized studies. The majority were conducted in Turkey (*n* = 9) and the United States (*n* = 6). Thirteen studies used 1,470-nm EVLA, while nine did not report wavelength data. Regarding fiber type, 18 studies (54.55%) did not specify the EVLA tip used; 12 (36.36%) used radial fibers, 2 (6.06%) used bare-tip, and 1 (3.03%) used ball-tip fibers.

**Table 1:** Baseline characteristics of included studies comparing RFA to EVLA in patients with chronic venous insufficiency.

Author (YOP)	Design	Country	Varicose Vein (%)	Interventions		Sample Size				Operated Side (%)		Bilaterality (%)		Age (year)				Gender (M/F)	
				EVLA	RFA	Patients		Limbs		EVLA	RFA	EVLA	RFA	EVLA		RFA		EVLA	RFA
						EVLA	RFA	EVLA	RFA	Rt/Lf	Rt/Lf			Mean	SD	Mean	SD		
Almeida (2009) (29)	RCT	Germany	90	980-nm	ClosureFAST	69		41	46	–	–	–	–	52.4	15.3	51.6	12.8	10/31	12/29
Almeida (2006) (30)	NRSI (retrospective)	USA	–	810, 940, 980, 1,320-nm	–	694		–	–	–	–	–	–	–	–	–	–	–	–
Atik (2024) (31)	NRSI (retrospective)	Turkey	–	–	–	412	230	–	–	–	–	–	–	46.24	11.31	41.56	10.22	98/314	74/183
Aurshina (2018) (7)	NRSI (retrospective)	New York	–	1,470-nm	ClosureFAST	1,027	448	–	–	736/739		–	–	74.6	9.8	61.1	14.9	–	–
Bozoglan (2016) (32)	NRSI (retrospective)	Turkey	–	1,470-nm	ClosureFAST	30	30	60	60	–	–	100	100	42.8 (10.0)				–	–
Chan (2024) (33)	NRSI (retrospective)	Philadelphia	–	–	–	3,556	11,605	–	–	–	–	–	–	55	13.6	54.4	13.7	1270/2286	3919/7686
Dzieciuchowicz (2014) (34)	RCT	Spain	–	810-nm	ClosureFAST	14	13	14	13	–	–	0	0	59.4	16.47	55.7	9.97	6/8	6/7
El Kilic (2022) (8)	NRSI (retrospective)	Turkey	–	1,470-nm	ClosureFAST	77	82	85	88	38/35	36/43	5.19	3.66	51.2	10.8	48.8	9.9	33/44	37/45
El Mahdi (2021) (9)	RCT	Egypt	100	–	–	10	10	–	–	3/7	7/3	0	0	35.6	6.35	27.2	6.6	6/4	4/6
Eroglu (2018) (35)	RCT	Turkey	–	1,470-nm	ClosureFAST	139	149	278	298	70/69	82/67	0	0	45.9	10.4	44.9	10.5	66/73	65/84
Gianesini (2021) (36)	NRSI (retrospective)	Italy	100	1,470-nm	ClosureFAST	38	41	41	44	–	–	5.26	7.32	54	10	56	10	14/24	16/25
Hamann (2019) (37)	RCT	Netherlands	–	980-nm	ClosureFAST (dRFA)	148	152	–	–	61/87	72/80	0	0	51.1	13.6	52.6	13.4	51/97	49/103
			–		ClosureFAST (iRFA)		150	–	–		71/79		0		13.6	49.3	13.9		45/105
Hemşinli (2020) (12)	NRSI (retrospective)	Turkey	100	1,470-nm	(Closure FAST)	86	271	–	–	–	–	–	–	46.06	10.15	43.35	10.08	42/44	113/158
Irungu (2021) (38)	NRSI (retrospective)	Kenya	–	–	ClosureFAST	139		139	263	–	–	89	89	–	–	–	–	38/101	
Kempeneers (2022) (39)	RCT	Belgium	–	1,470-nm	ClosureFAST	142	138	–	–	–	–	–	–	51.48	12.883	51.54	13.173	48/94	55/83
Korepta (2022) (13)	NRSI (retrospective)	Spain	26	–	–	84	46	–	–	–	–	–	–	63.68	14.51	63.76	13.92	46/38	25/21
Korkmaz (2017) (40)	NRSI (retrospective)	Turkey	100	1,470-nm	ClosureFAST	161	124	–	–	–	–	–	–	–	–	–	–	70/91	50/74
				980-nm		95	124	–	–					–	–	–	–	47/48	
Kubat (2020) (11)	NRSI (retrospective)	Turkey	14.6	980-nm	ClosureFAST	39	121	–	–	108/146		5.2		44.54	13.62	45.79	12.16	23/16	55/66
				1,470-nm		36		–	–					44	12.97			21/15	
Kubat (2021) (10)	NRSI (retrospective)	Turkey	100	980-nm	ClosureFAST	151	264	–	–	–	–	–	–	48.8	10.4	49.5	11.4	62/86	118/131
			–	1,470-nm		109		–	–			–	–	47.4	11.4			44/65	
Lawson (2018) (41)	NRSI (prospective)	Netherlands	100	–	–	153	158	171	175	–	–	–	–	50	13.75	49.9	13.57	43/110	38/120
Marston (2006) (42)	NRSI (retrospective)	USA	–	810-nm	ClosureFAST	80		31	58	–	–	–	–	57.6	14.9	52.5	11.6	–	18/40
Mubarak (2023) (43)	RCT	Egypt	100	–	ClosureFAST	20	20	–	–	–	–	–	–	–	–	–	–	–	–
Nordon (2011) (44)	RCT	UK	100	810-nm	ClosureFAST	80	79	–	–	–	–	–	–	46.7	14.4	46.9	15.1	26/54	34/45
Park (2020) (18)	NRSI (prospective)	South Korea	100	1,940-nm	ClosureFAST	43	37	–	–	–	–	–	–	47.4	13.8	40.4	14.3	13/30	17/25
Park (2023) (46)	NRSI (retrospective)	South Korea.	100	–	ClosureFAST	48	43	51	43	–	–	6.25	0	57.2	14.5	60.3	12.3	19/29	20/23
Prabakar (2020) (47)	NRSI (retrospective)	India	100	1,470-nm	ClosureFAST	50	50	-	-	-	-	-	-	49	-	47	-	-	-
Ravi (2006) (49)	NRSI (retrospective)	USA	91.9	940-nm	ClosureFAST	-	-	1,091	159	604/646		-	-	-	-	-	-	-	-
Ravi (2009) (48)	NRSI (retrospective)	USA	-	940-nm	ClosureFAST	-	-	2,841	159	-	-	-	-	-	-	-	-	-	-
Reem (2023) (50)	NRSI (prospective)	Egypt	–	–	ClosureFAST	65	65	–	–	–	–	–	–	32.86	6.34	33.28	5.29	8/57	10/55
Sydnor (2017) (51)	RCT	USA	100	980-nm	ClosureFAST	100	100	–	–	53/47	47/53	0	0	48.5	10.5	47	11.2	23/77	20/80
Tuan (2020) (52)	NRSI (retrospective)	Vietnam	80.4	1,470-nm	–	41	8	–	–	–	–	–	–	–	–	–	–	–	–
Yoon (2019) (19)	NRSI (retrospective)	USA	48.8	810-nm	ClosureFAST	183	87	246	97	–	–	12.6	5.43	56.6	12.4	59.8	15.3	49/134	32/55
Öntas (2019) (45)	NRSI (retrospective)	Turkey	100	1,470-nm	ClosureFAST	25	25	25	25	–	–	0	0	–	–	–	–	–	-

YOP, Year of Publication; RCT, Randomized Controlled Trial; NRSI, Non-randomized Studies of Intervention; EVLA, Endovenous Laser Ablation; RFA, Radiofrequency Ablation; M/F, Male/Female; Rt/Lf, Right/Left; SD, Standard Deviation.

Across all studies, 22,814 patients with CVI were included—8,144 treated with EVLA and 14,670 with RFA. The unit of analysis was the patient rather than the treated limb. In studies reporting bilateral procedures, outcomes were extracted at the patient level whenever available; studies reporting limb-level outcomes without patient-level aggregation were not pooled to avoid clustering effects. Additional demographic and clinical data (e.g., age, gender, laterality, and varicose vein status) are presented in Supplementary Table S3.

### 3.3 Risk of bias results

**Figure 2:**
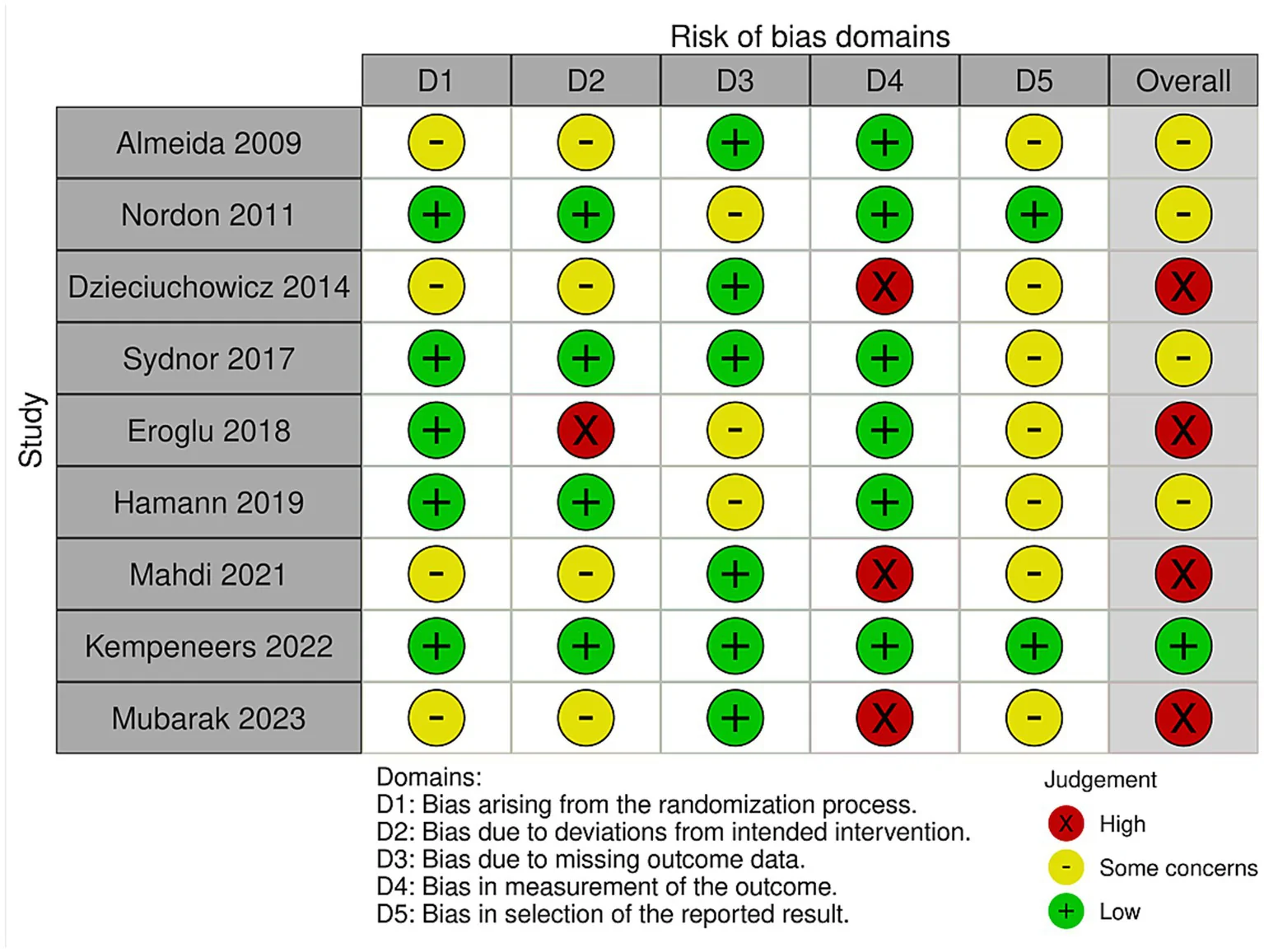
Risk-of-bias assessment of the included randomized controlled trials using the revised Cochrane risk-of-bias tool (RoB 2). The traffic-light plot shows the judgement (low risk, some concerns, or high risk) for each individual trial across the five RoB 2 domains—randomization process, deviations from intended interventions, missing outcome data, measurement of the outcome, and selection of the reported result—together with the overall risk-of-bias rating for each trial, allowing the basis for every judgement to be inspected directly.

Among the nine included randomized controlled trials, only one study was judged to have an overall low risk of bias, while the remaining eight raised some concerns (4 trials) or were at high risk (4 trials). Figure 2 presents the domain-level (traffic-light) judgements for every trial across all five RoB-2 domains—randomization, deviations from intended interventions, missing outcome data, outcome measurement, and selection of the reported result—together with the overall rating, allowing the basis for each judgement to be inspected directly. Because most trials were not at low risk of bias, these ratings were carried through to the interpretation of every pooled estimate. The most frequent sources of bias were related to the randomization process and selective reporting, which were commonly rated as some concerns. High risk of bias was observed mainly in the outcome measurement domain, whereas bias due to missing outcome data was generally low across trials.

Among the 24 observational studies, 18 studies were of good quality, three were of fair quality, and three were of poor quality (Table 2).

**Table 2:** A summary of the methodological quality of non-randomized studies of intervention comparing endovenous to radiofrequency ablation using the Newcastle Ottawa Scale.

Study ID	Selection				Comparability	Outcome			Overall scoring	Overall rating
	Is the case definition adequate/ Representativeness of the exposed cohort	Representativeness of the cases/Selection of the non-exposed cohort	Selection of controls/ascertainment of exposure	Definition of controls/demonstration that outcome of interest was not present at start of study	Comparability of cases and controls (cohorts) on the basis of the design or analysis	Ascertainment of exposure/assessment of outcome	Same method of ascertainment for cases and controls/ Was follow-up long enough for outcomes to occur	Non-response rate/adequacy of follow up of cohorts		
Almeida (2006) (30)	1	1	1	0	1	1	1	0	3/1/2	Good
Atik (2024) (31)	1	1	1	0	1	1	1	1	3/1/3	Good
Aurshina (2018) (7)	1	1	1	0	1	1	1	0	3/1/2	Good
Bozoglan (2016) (32)	0	0	1	0	1	1	1	1	1/1/3	Poor
Chan (2024) (33)	1	1	1	0	1	1	0	0	3/1/1	Fair
El Kilic (2022) (8)	0	0	1	1	1	1	1	1	3/1/3	Good
Gianesini (2021) (36)	1	1	1	0	1	1	1	1	3/1/3	Good
Hemşinli (2020) (12)	1	1	0	0	1	1	1	0	2/1/2	Fair
Irungu (2021) (38)	0	0	1	1	1	1	1	1	3/1/3	Good
Korepta (2022) (13)	1	1	1	0	1	1	1	0	3/1/2	Good
Korkmaz (2017) (40)	1	1	1	0	1	1	0	0	3/1/1	Fair
Kubat (2020) (11)	1	1	1	0	1	1	1	0	3/1/2	Good
Kubat (2021) (10)	1	1	1	0	1	1	1	0	3/1/2	Good
Lawson (2018) (41)	1	1	1	0	1	1	1	0	3/1/2	Good
Marston (2006) (42)	1	1	1	0	1	1	1	1	3/1/3	Good
Park (2020) (18)	1	0	1	1	1	1	1	0	3/1/2	Good
Park (2023) (46)	1	1	1	0	1	1	1	1	3/1/3	Good
Ravi (2006) (48)	1	1	1	0	1	1	1	0	3/1/2	Good
Ravi (2009) (49)	1	1	1	0	1	1	1	0	3/1/2	Good
Reem (2023) (50)	0	0	1	0	1	1	1	1	1/1/3	Poor
Tuan (2020) (52)	1	0	1	1	1	1	1	0	3/1/2	Good
Yoon (2019) (19)	1	1	1	0	1	1	1	1	3/1/3	Good
Öntas (2019) (45)	0	0	1	0	1	1	1	1	1/1/3	Poor

### 3.4 Efficacy outcomes

#### 3.4.1 Success rate

Three non-randomized studies (502 patients) assessed procedural success (Supplementary Figure S1). Subgroup analysis showed no effect modification by EVLA wavelength (*p* = 0.22) or follow-up duration (*p* = 0.30). No significant difference was observed between EVLA and RFA overall (OR = 0.92; 95% CI: 0.38–2.25), regardless of wavelength. A single study at 36 months reported higher success with RFA (OR = 3.06; 95% CI: 1.12–8.37). Meta-regression revealed no significant covariates; model fit was poor (*R*^2^ = 19.22%) with low heterogeneity (*I*^2^ = 23.99%) (Table 3).

**Table 3:** Meta-regression analysis of the determinants of analyzed outcomes in patients with chronic venous insufficiency undergoing either RFA or EVLA.

Covariate	Success rate		Recanalization rate		Complete occlusion		Partial occlusion	
	Coefficient	*p*	Coefficient	*p*	Coefficient	*p*	Coefficient	*p*
RCT (vs. NRSI)	0.000	Omitted	0.000	Omitted	−0.533	0.395	0.161	0.878
EVLA wavelength [Reference: 810-nm]								
980 nm	-	-	0.719	0.703	−1.809	**0.031**	0.152	0.892
1,470 n m	−0.593	0.544	−0.085	0.954	−2.852	**0.0001**	0.402	0.644
1940 nm	−1.029	0.447	-	-	-	-	-	-
940 nm	-	-	-	-	-	-	0.569	0.228
Follow-up (per month)	0.022	0.331	0.040	0.735	0.010	0.560	−0.011	0.544
Residual Heterogeneity (*I*^2^)	23.990		84.230		90.920		48.910	
Model Fit (*R*^2^)	19.220		0		0		0	

	Recurrence rate		Reflux-free rate		Pain score		VCSS score	
	Coefficient	*p*	Coefficient	*p*	Coefficient	*p*	Coefficient	*p*
Severe CVI [CEAP 4–6 vs. 1–3]	-	-	-	-	0.570	**0.0001**	0.017	**0.043**
RCT (vs. NRSI)	1.886	0.123	0.000	Omitted	−4.046	**0.003**	−0.161	0.242
EVLA wavelength [Reference: 810-nm]								
980 nm	−1.346	**0.011**	-	-	−1.173	0.189	0.237	0.140
1,470 nm	−0.471	0.126	1.037	0.553	−3.845	**0.0001**	0.000	Omitted
1,940 nm	0.000	Omitted	−0.313	0.827	0.000	Omitted	-	-
940 nm	0.000	Omitted	-	-	-	-	-	-
Follow-up (per month)	−0.021	0.155	−0.021	0.841	0.172	**0.007**	−0.005	**0.004**
Residual Heterogeneity (*I*^2^)	5.200		100		94.920		12.790	
Model Fit (*R*^2^)	0		0		49.550		80.720	

	AVVQ score		GSV diameter		Time to return to daily activities	
	Coefficient	*p*	Coefficient	*p*	Coefficient	*p*
Severe CVI [CEAP 4–6 vs. 1–3]	−0.255	0.606	0.000	Omitted	−0.790	**0.0001**		
RCT (vs. NRSI)	−0.173	0.834	0.000	Omitted	11.117	**0.0001**		
EVLA wavelength [Reference: 810-nm]								
980 nm	0.000	Omitted	0.000	Omitted	0.000	Omitted		
1,470 n m	0.000	Omitted	-	-	0.000	Omitted		
1,940 nm	-	-	-	-	-	-		
Follow-up (per month)	0.012	0.629	−0.106	**0.011**	-	-		
Residual Heterogeneity (*I*^2^)	38.470		80.340		0			
Model Fit (*R*^2^)	0		77.270		0			

Variables labeled as “omitted” or “not applicable” indicate covariates that could not be included in the corresponding meta-regression model due to insufficient reporting across studies, lack of variability within the analyzed subgroup, or collinearity leading to model non-convergence. Only covariates reported by at least five studies and meeting predefined eligibility criteria were included in meta-regression analyses. RFA: Radiofrequency Ablation; EVLA: Endovenous Laser Ablation; P: *p*-value; GSV: Great Saphenous Vein; CVI: Chronic Venous Insufficiency; RCT: Randomized Controlled Trial; NRSI: Non-Randomized Studies of Intervention; VCSS: Venous Clinical Severity Score; AVVQ: Aberdeen Varicose Vein Questionnaire. Bold values indicate statistical significance at *p* < 0.05.

#### 3.4.2 Recanalization rate

Seven non-randomized studies (3,977 patients) reported on recanalization (Supplementary Figure S2). No significant differences were found between RFA and EVLA (OR = 1.79; 95% CI: 0.73–4.35), and neither EVLA wavelength (*p* = 0.56) nor follow-up period (*p* = 0.49) were significant modifiers. However, one study found higher recanalization with RFA compared to 980-nm EVLA (OR = 3.33; 95% CI: 1.32–8.41). No such difference was observed for 810 or 1,470-nm. Meta-regression identified no significant covariates, and model fit was poor (*R*^2^ = 0%) with high heterogeneity (*I*^2^ = 84.23%). (Table 3).

#### 3.4.3 Complete occlusion rate

**Figure 3:**
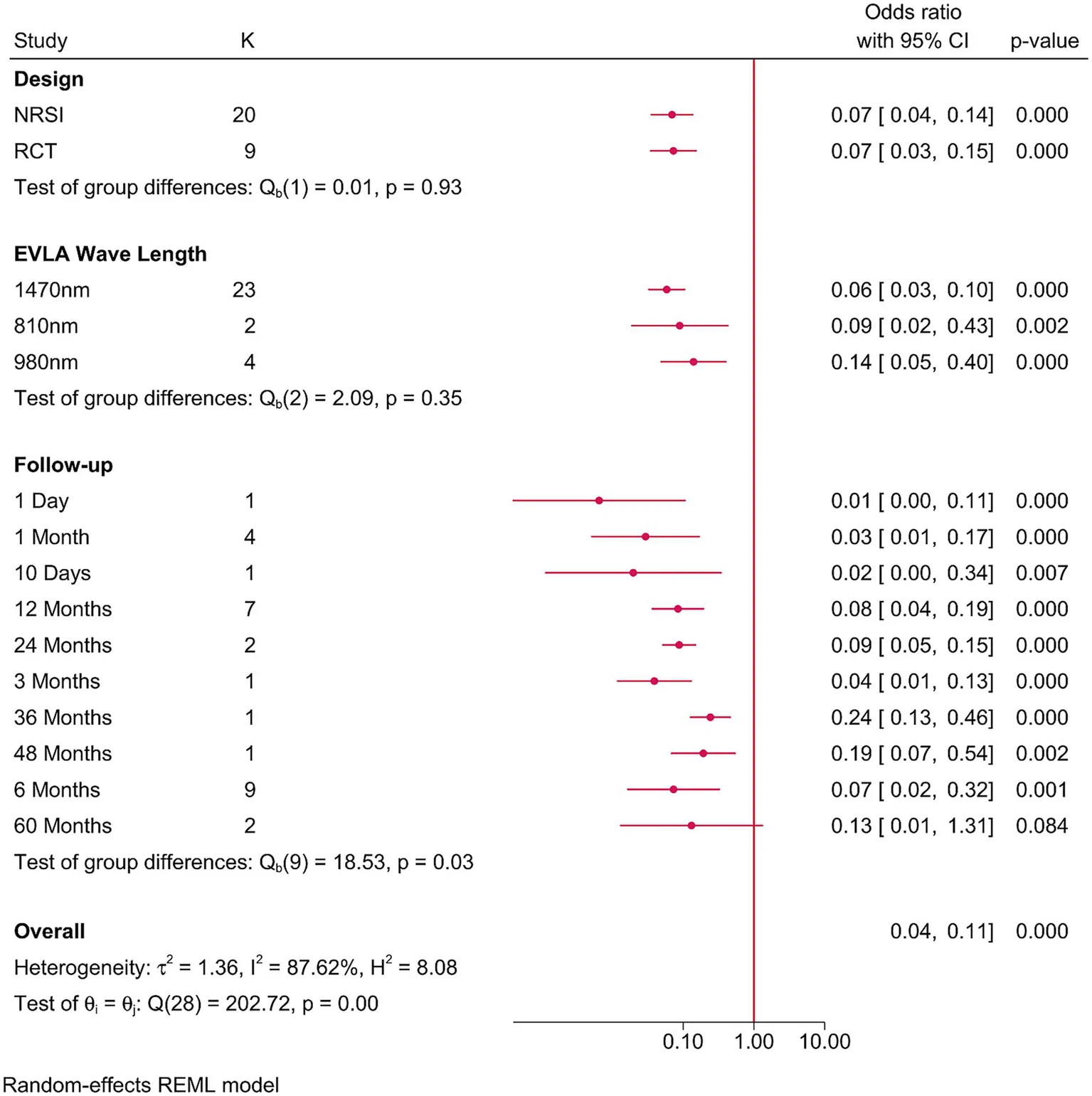
Forest plot comparing radiofrequency ablation (RFA) with endovenous laser ablation (EVLA) for complete occlusion of the treated vein (the primary outcome) in patients with chronic venous insufficiency. Effect estimates are odds ratios (OR) with 95% confidence intervals, pooled using a random-effects model (REML method), with EVLA as the reference group; results are stratified by study design (randomized vs. non-randomized), EVLA wavelength, and follow-up time. Each square represents the OR of an individual study (square size proportional to its weight in the analysis), the horizontal line its 95% confidence interval, and each diamond the pooled subgroup or overall estimate. An OR below 1 indicates lower odds of complete occlusion with RFA relative to EVLA.

Fifteen studies (3 RCTs and 12 NRSIs; 3,478 patients) evaluated complete occlusion, the prespecified primary outcome (Figure 3). Follow-up time was a significant effect modifier (*p* = 0.03). RFA was associated with lower odds of complete occlusion in both RCTs (OR = 0.07; 95% CI: 0.04–0.14) and NRSIs (OR = 0.07; 95% CI: 0.03–0.15), particularly at early follow-up (1 day: OR = 0.01; 95% CI: 0.00–0.11). The magnitude of this early effect should be interpreted cautiously, as definitions of complete occlusion and imaging timing varied across studies (e.g., residual flow thresholds and duplex protocols). The difference decreased over time and became non-significant by 60 months (OR = 0.13; 95% CI: 0.01–1.31). The effect persisted across EVLA wavelengths.

Meta-regression showed EVLA wavelength as a significant predictor, with 1,470-nm EVLA outperforming 810-nm EVLA (coefficient = −2.85; *p* = 0.00001). Model fit remained poor (*R*^2^ = 0%) with high heterogeneity (*I*^2^ = 90.92%) (Table 3).

#### 3.4.4 Partial occlusion rate

Five studies (2 RCTs, 3 NRSIs; 2,155 patients) assessed partial occlusion (Supplementary Figure S3). No significant differences were found between EVLA and RFA across study design, wavelength, or follow-up (*p*-values: 0.77, 0.75, and 0.53, respectively). Meta-regression identified no effect-modifying covariates. Model fit was poor (*R*^2^ = 0%) with moderate heterogeneity (*I*^2^ = 48.91%) (Table 3).

### 3.5 Patient-reported and safety outcomes

#### 3.5.1 Reflux-free rate

Three non-randomized studies (570 patients) were included (Supplementary Figure S4). No significant differences between RFA and EVLA were found, and EVLA wavelength (*p* = 0.78) and follow-up duration (*p* = 0.93) were not effect modifiers. Meta-regression found no significant covariates (*R*^2^ = 0%, *I*^2^ = 100%) (Table 3).

#### 3.5.2 Postoperative pain

**Figure 4:**
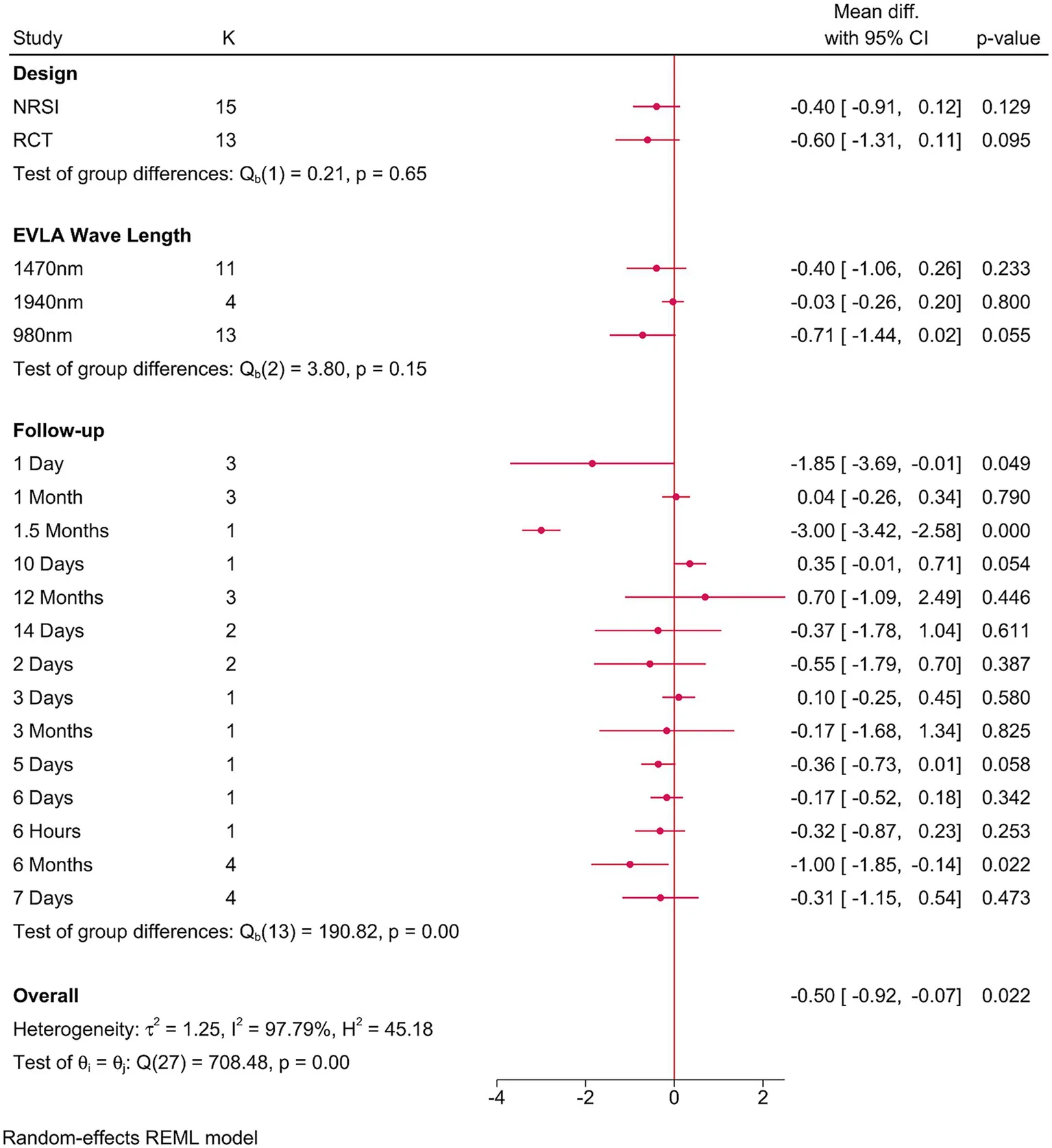
Forest plot comparing radiofrequency ablation (RFA) with endovenous laser ablation (EVLA) for postoperative pain score in patients with chronic venous insufficiency. Effect estimates are mean differences (MD) with 95% confidence intervals, pooled using a random-effects model (REML method), with EVLA as the reference group; results are stratified by study design (randomized vs. non-randomized), EVLA wavelength, and follow-up time. Each square represents the MD of an individual study (square size proportional to its weight in the analysis), the horizontal line its 95% confidence interval, and each diamond the pooled subgroup or overall estimate. An MD below 0 indicates lower postoperative pain with RFA relative to EVLA.

Fourteen studies (5 RCTs and 9 NRSIs; 2,647 patients) reported postoperative pain, primarily measured using a 0–10 Visual Analogue Scale (VAS). While overall pooled pain scores did not differ significantly between groups, RFA was associated with lower pain at 1 day (MD = −1.85; 95% CI: −3.69 to −0.01), 6 weeks (MD = −3.00; 95% CI: −3.42 to −2.58), and 6 months (MD = −1.00; 95% CI: −1.85 to −0.14) (Figure 4).

Meta-regression showed pain was associated with more severe CVI (*p* = 0.0001), longer follow-up (*p* = 0.007), and reduced with 1,470-nm EVLA (p = 0.0001). Model fit was moderate (*R*^2^ = 49.55%, *I*^2^ = 94.92%) (Table 3).

#### 3.5.3 Postoperative VCSS score

**Figure 5:**
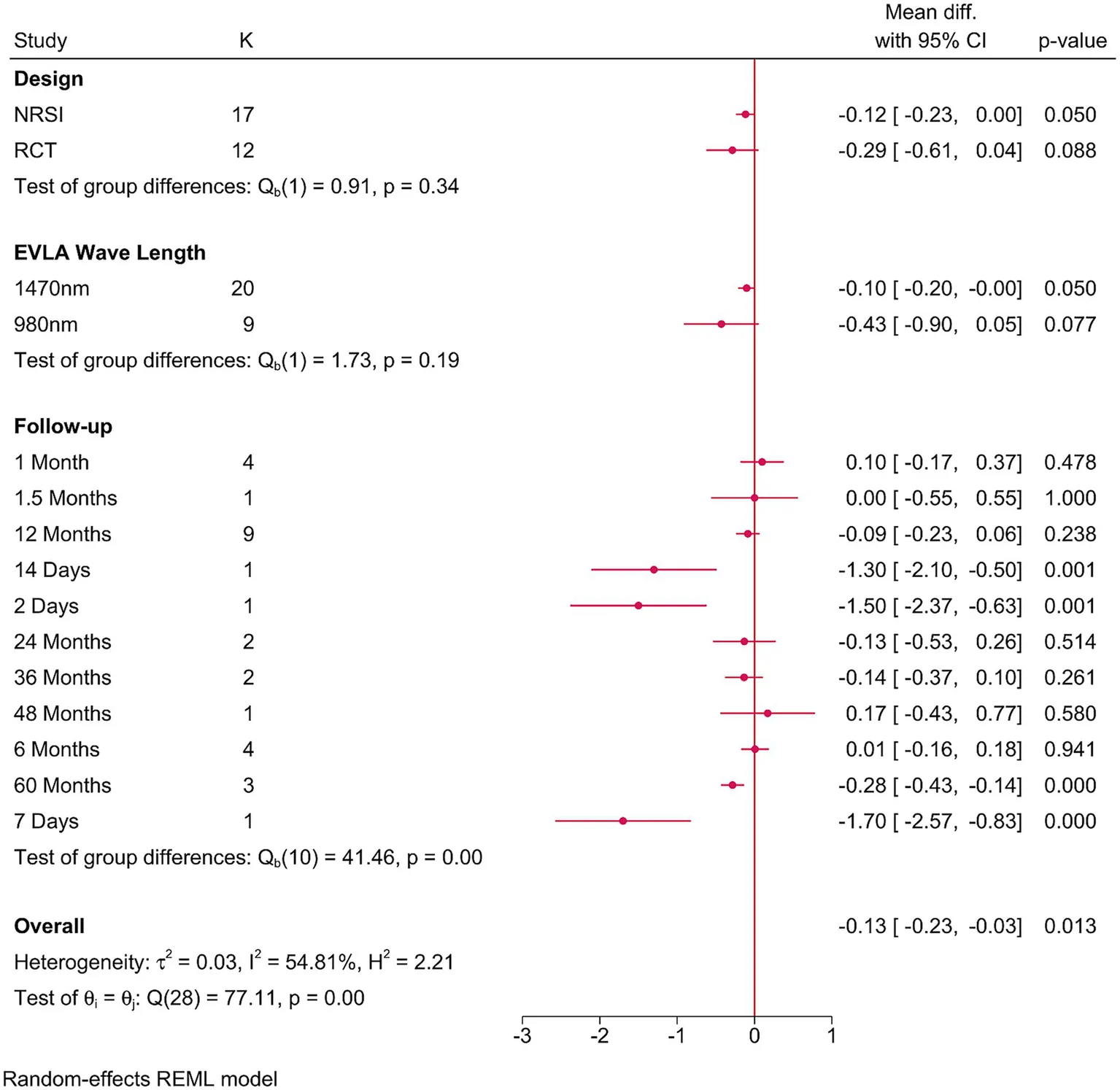
Forest plot comparing radiofrequency ablation (RFA) with endovenous laser ablation (EVLA) for postoperative Venous Clinical Severity Score (VCSS) in patients with chronic venous insufficiency. Effect estimates are mean differences (MD) with 95% confidence intervals, pooled using a random-effects model (REML method), with EVLA as the reference group; results are stratified by study design (randomized vs. non-randomized), EVLA wavelength, and follow-up time. Each square represents the MD of an individual study (square size proportional to its weight in the analysis), the horizontal line its 95% confidence interval, and each diamond the pooled subgroup or overall estimate. An MD below 0 indicates a lower (better) VCSS with RFA relative to EVLA.

Eleven trials (4 RCTs and 7 NRSIs; 2,675 patients) were analyzed. Follow-up duration was the only significant modifier of postoperative VCSS score (*p* = 0.001) (Figure 5). Overall postoperative VCSS scores were comparable between RFA and EVLA across study designs and wavelengths (980-nm and 1,470-nm). However, RFA was associated with lower VCSS scores during early postoperative follow-up (2, 7, and 14 days), based on single-study estimates.

Meta-regression showed VCSS scores decreased with longer follow-up (coefficient = −0.005; *p* = 0.004). Model fit was good (*R*^2^ = 80.72%) with low residual heterogeneity (*I*^2^ = 12.79%) (Table 3).

#### 3.5.4 Postoperative AVVQ score

Four trials (2 RCTs and 2 NRSIs; 855 patients) were analyzed (Supplementary Figure S5). Study design, EVLA wavelength, and follow-up period were not significant effect modifiers. No significant differences were observed between RFA and EVLA across all subgroups. Meta-regression identified no significant determinants, with moderate residual heterogeneity (*R*^2^ = 0%, *I*^2^ = 38.47%).

#### 3.5.5 Postoperative GSV diameter

**Figure 6:**
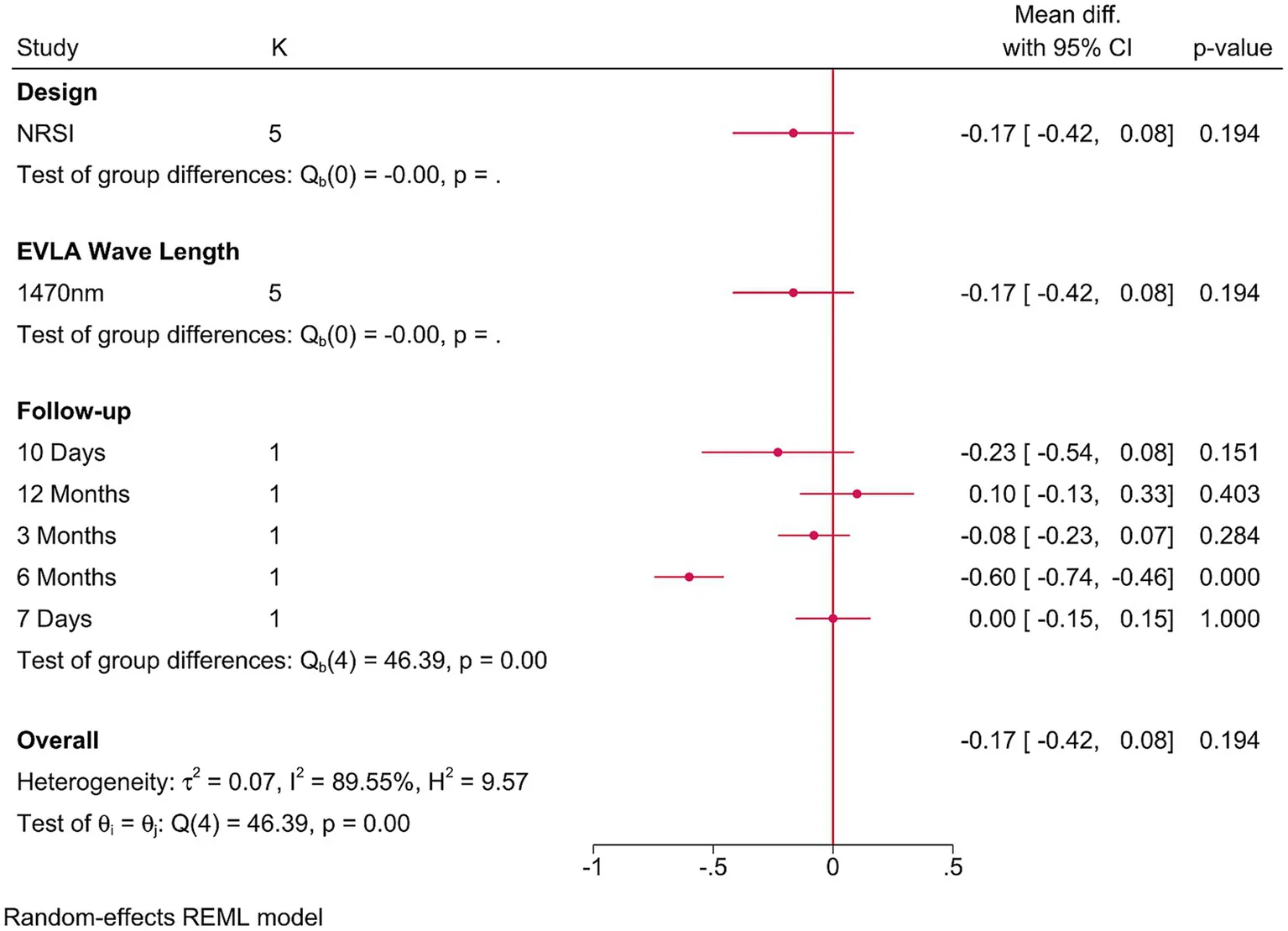
Forest plot comparing radiofrequency ablation (RFA) with endovenous laser ablation (EVLA) for postoperative great saphenous vein (GSV) diameter in patients with chronic venous insufficiency. Effect estimates are mean differences (MD) with 95% confidence intervals, pooled using a random-effects model (REML method), with EVLA as the reference group; results are stratified by study design (randomized vs. non-randomized), EVLA wavelength, and follow-up time. Each square represents the MD of an individual study (square size proportional to its weight in the analysis), the horizontal line its 95% confidence interval, and each diamond the pooled subgroup or overall estimate. An MD below 0 indicates a smaller GSV diameter with RFA relative to EVLA.

Three non-randomized studies (265 patients) were analyzed. Follow-up duration was the only significant modifier (*p* = 0.001) (Figure 6). No overall difference between RFA and EVLA was observed (MD = −0.17; 95% CI: −0.42 to 0.08). However, RFA showed smaller GSV diameter at 6 months postoperatively (MD = −0.60; 95% CI: −0.74 to −0.46).

Meta-regression showed GSV diameter decreased with longer follow-up (coefficient = −0.106; *p* = 0.011). Model fit was good (*R*^2^ = 77.27%) with high residual heterogeneity (*I*^2^ = 80.34%) (Table 3).

#### 3.5.6 Time to return to work (day)

Three trials (1 RCT and 2 NRSIs; 500 patients) were analyzed (Supplementary Figure S6). Study design significantly modified treatment effects (*p* = 0.03). Non-randomized studies suggested a longer time to return to work with RFA (MD = 0.49 days; 95% CI: 0.10–0.88), whereas the randomized trial showed no difference (MD = −0.87 days; 95% CI: −2.05 to 0.31). Only 1,470-nm EVLA was evaluated.

#### 3.5.7 Time to return to daily activities (day)

Five trials (1 RCT and 4 NRSIs; 739 patients) were analyzed (Supplementary Figure S7). No differences were observed between RFA and EVLA across study designs or wavelengths. Severe CVI was associated with faster return to daily activities (coefficient = −0.790; *p* = 0.0001), whereas RCT design was associated with longer recovery periods (coefficient = 11.11; p = 0.0001) (Table 3). Model fit was poor (*R*^2^ = 0%) with no residual heterogeneity (*I*^2^ = 0%).

#### 3.5.8 Satisfaction score

Three trials (2 RCTs, 1 NRSI), including 558 patients, were analyzed (Supplementary Figure S8). No significant differences between RFA and EVLA were observed across study designs, EVLA wavelengths, or follow-up durations.

#### 3.5.9 Recurrence rate

Seven trials (1 RCT and 6 NRSIs; 3,849 patients) were analyzed (Supplementary Figure S9). Non-randomized studies suggested lower recurrence with RFA (OR = 0.42; 95% CI: 0.29–0.62), including comparisons with 980-nm EVLA and 1,470-nm EVLA. However, randomized evidence showed no significant difference between treatments. Most observational data were limited to short follow-up (≤12 months).

Meta-regression identified EVLA wavelength as the only determinant (coefficient = −1.34; *p* = 0.011), with low residual heterogeneity (*I*^2^ = 5.20%) but poor model fit (*R*^2^ = 0%).

#### 3.5.10 Complications

The meta-analysis showed no difference in the risk of postoperative complications between RFA and EVLA (OR = 0.72; 95%CI: 0.44, 1.19) (Supplementary Figure S10). This lack of difference was maintained across all of the examined time points (represented by at least 2 studies to provide reliable findings). Reported complications included paresthesia, infection, induration, hyperpigmentation, ecchymosis, deep vein thrombosis (DVT), and phlebitis (Table 4). No differences in the risk of complications between RFA and EVLA were noted across all time points in terms of infection, induration, hyperpigmentation, DVT, and phlebitis. Meanwhile, RFA was associated with a lower risk of paresthesia at 1 month (OR = 0.52; 95%CI: 0.28–0.95) and ecchymosis at 1 week (OR = 0.25; 95%CI: 0.10–0.62) and 12 months (OR = 0.27; 95%CI: 0.13–0.57). Although Table 4 shows additional statistically significant estimates for paresthesia at 60 months and ecchymosis at 2 days, 2 weeks, and 24 months, each of these estimates was based on a single study and was therefore not highlighted as a reliable finding in the narrative.

**Table 4:** The risk of different complications reported in patients with CVI undergoing RFA versus EVLA.

Follow-up	Paresthesia		Infection		Induration		Hyperpigmentation		Ecchymosis		DVT		Phlebitis	
	OR	95% CI	OR	95% CI	OR	95% CI	OR	95% CI	OR	95% CI	OR	95% CI	OR	95% CI
1 Day	1.57	0.77–3.19	0.65	0.11–4.00	–	–	0.99	0.19–5.03	0.39	0.07–2.05	–	–	–	–
2 Days	0.29	0.01–7.33	0.89	0.02–45.99	–	-	0.89	0.02–45.99	0.12	0.04–0.32	–	–	11	0.59–205.36
1 Week	2.74	0.11–69.04	0.89	0.02–45.99	–	–	0.89	0.02–45.99	0.25	0.10–0.62	–	–	2.74	0.11–69.04
2 Weeks	0.42	0.04–4.85	0.89	0.02–45.99	–	–	0.89	0.02–45.99	0.12	0.05–0.33	–	–	–	–
1 Month	0.52	0.28–0.95	1.08	0.07–17.41	0.84	0.19–3.76	0.77	0.28–2.07	0.64	0.28–1.49	0.56	0.13–2.42	0.82	0.41–1.64
1.5 Months	1.5	0.61–3.68	–	–	–	–	–	–	–	–	0.99	0.02–50.39	–	–
3 Months	1.01	0.17–5.91	1	0.02–55.27	–	–	1	0.02–55.27	0.16	0.01–3.85	1	0.02–55.27	–	–
6 Months	1	0.02–51.22	–	–	1.85	0.80–4.27	1.74	0.83–3.65	0.82	0.53–1.27	0.68	0.07–6.61	–	–
12 Months	0.54	0.22–1.36	–	–	0.6	0.02–14.67	0.4	0.07–2.27	0.27	0.13–0.57	0.73	0.17–3.19	1.79	0.04–90.49
24 Months	–	–	–	–	–	–	–	–	4.91	1.96–12.29	2.82	0.11–69.76	–	–
60 Months	0.35	0.13–0.97	–	–	1.79	0.04–90.49	0.39	0.09–1.74	1.79	0.04–90.49	0.49	0.07–3.50	–	–

RFA, Radiofrequency Ablation; EVLA, Endovenous Laser Ablation; CI, Confidence Interval; OR, Odds Ratio; CVI, Chronic Venous Insufficiency; DVT, Deep Vein Thrombosis.

## 4 Discussion

This systematic review and meta-analysis pooled 33 comparative studies (9 randomized trials and 24 non-randomized studies; 22,814 patients) to compare EVLA and RFA for CVI across technical and patient-centered outcomes. The central finding is straightforward: when either modality is performed competently, EVLA and RFA are clinically equivalent for the outcomes that matter most to patients—reflux-free status, quality of life, satisfaction, and functional recovery. The differences we detected were confined to early technical measures and short-term comfort and did not carry through to patient-relevant benefit. This is concordant with previous systematic reviews, which have likewise reported no meaningful difference between the two techniques.

On the primary outcome, complete occlusion, the only notable difference was a lower early occlusion rate with RFA, driven almost entirely by the 1-day time point. This early difference is best explained by between-study variation in occlusion definitions (e.g., residual-flow thresholds) and in the timing of duplex imaging rather than by a durable difference in efficacy; mechanistically, EVLA—particularly at 1470 nm, which targets water absorption—may produce more uniform early vein-wall injury, whereas RFA delivers segmental, temperature-controlled energy that is highly reproducible but can be less effective in large-diameter or tortuous veins (53, 54). Critically, the early advantage attenuated over time and did not translate into any difference in reflux-free status or patient-reported outcomes, so the day-1 occlusion difference is of little clinical importance (55).

Among secondary outcomes, RFA was associated with lower postoperative pain at 6 weeks and 6 months—a magnitude generally regarded as clinically meaningful—and with fewer cases of paresthesia and ecchymosis, consistent with prior reports of reduced pain and faster short-term recovery with RFA (32, 56, 57). These advantages did not, however, translate into differences in patient satisfaction, return to work, or return to daily activities (58), and major complications (deep vein thrombosis, hyperpigmentation, phlebitis) did not differ between modalities. Short-term comfort is therefore the main respect in which the two techniques diverge.

These findings must be read against the quality of the underlying evidence. Only one of the nine randomized trials was at low overall risk of bias, while the remainder raised some concerns or were at high risk—most often for the randomization process, deviations from intended interventions, or selective reporting; the domain-level RoB-2 judgements for every trial are shown in Figure 2. Results supported only by non-randomized data—most notably the lower recurrence rate with RFA, which was not reproduced in the randomized trials—should therefore be interpreted with caution.

EVLA wavelength was the principal modifier of technical occlusion, with 1,470-nm EVLA outperforming earlier wavelengths, in keeping with current practice (59). Even so, this technical advantage did not confer better reflux-free or quality-of-life outcomes, and substantial residual heterogeneity—attributable to inconsistently reported technical factors such as energy dosing, fiber type, pullback speed, operator experience, and imaging protocols—limits the strength of any modality-level comparison (60, 61).

Taken together, our results align with the existing literature in showing no clinically meaningful advantage of one thermal modality over the other for patient-centered outcomes (62). In practice, the choice between EVLA and RFA can reasonably be guided by operator familiarity, device availability, and patient preference rather than by an expectation of superior efficacy.

### 4.1 Study limitations

This analysis has several limitations. Substantial heterogeneity was observed across multiple outcomes, particularly in non-randomized studies. Although we accounted for study design, follow-up duration, and EVLA wavelength, residual heterogeneity persisted. This variability likely reflects differences in outcome definitions, duplex imaging protocols, residual flow thresholds, and technical parameters such as energy settings, fiber type, pullback speed, and radiofrequency catheter generation, which were inconsistently reported. Because more than half of the included EVLA studies did not specify the type of laser fiber used, it was not possible to evaluate whether differences in fiber design may have influenced treatment outcomes, which may limit the generalizability of the pooled EVLA results. Despite conducting meta-regression analyses, these variables explained little of the between-study variance, suggesting that additional unmeasured methodological and technical differences between studies likely contributed to the substantial heterogeneity observed.

Variability in outcome definitions and imaging timing may have particularly influenced early occlusion estimates. Differences in operator experience, institutional procedural volume, and peri-procedural protocols—including tumescent anesthesia technique, adjunctive treatments, and compression strategies—may also have contributed to observed heterogeneity.

The unit of analysis was the patient rather than the treated limb; however, some primary studies reported bilateral procedures or limb-level outcomes, which may introduce clustering effects that could not be fully addressed. Incomplete reporting also limited evaluation of long-term recurrence and quality-of-life outcomes. The observed reduction in recurrence with RFA was based largely on observational data with short follow-up and was not confirmed in randomized trials.

Publication bias could not be formally assessed for most outcomes because few analyses included sufficient studies. Cost-effectiveness was not evaluated, and emerging device generations (e.g., newer EVLA wavelengths or RFA systems) may influence future comparative effectiveness. Given ongoing technological developments, periodic updates or a living systematic review approach may be warranted (62).

## 5 Conclusion

This systematic review and meta-analysis demonstrate that both EVLA and RFA are effective and safe treatments for CVI. While EVLA—particularly with 1,470-nm wavelengths—achieves more consistent early technical occlusion, RFA provides short-term benefits in postoperative comfort and selected complications. These technical differences do not translate into clear differences in reflux-free status or patient-reported quality-of-life outcomes, and our results are concordant with previous meta-analyses reporting no meaningful difference between the modalities. Future studies should prioritize high-quality randomized evidence and patient-reported outcomes to better guide individualized treatment selection. Overall, when either treatment is performed competently, neither modality confers a clinically meaningful advantage over the other for patient-centered outcomes, so treatment selection can reasonably be guided by anatomical considerations, operator familiarity, and patient preference rather than by a presumed difference in efficacy.

## Data Availability

The original contributions presented in the study are included in the article/Supplementary material, further inquiries can be directed to the corresponding author.

## Supplementary material

FIGURE S1 Forest plot comparing radiofrequency ablation (RFA) with endovenous laser ablation (EVLA) for procedural success rate in patients with chronic venous insufficiency. Effect estimates are odds ratios (OR) with 95% confidence intervals, pooled using a random-effects model (REML method), with EVLA as the reference group; results are stratified by study design (randomized vs. non-randomized), EVLA wavelength, and follow-up time. Each square represents the OR of an individual study (square size proportional to its weight in the analysis), the horizontal line its 95% confidence interval, and each diamond the pooled subgroup or overall estimate. An OR above 1 indicates higher odds of procedural success with RFA relative to EVLA.

FIGURE S2 Forest plot comparing radiofrequency ablation (RFA) with endovenous laser ablation (EVLA) for recanalization rate in patients with chronic venous insufficiency. Effect estimates are odds ratios (OR) with 95% confidence intervals, pooled using a random-effects model (REML method), with EVLA as the reference group; results are stratified by study design (randomized vs. non-randomized), EVLA wavelength, and follow-up time. Each square represents the OR of an individual study (square size proportional to its weight in the analysis), the horizontal line its 95% confidence interval, and each diamond the pooled subgroup or overall estimate. An OR above 1 indicates higher odds of recanalization with RFA relative to EVLA.

FIGURE S3 Forest plot comparing radiofrequency ablation (RFA) with endovenous laser ablation (EVLA) for partial occlusion rate in patients with chronic venous insufficiency. Effect estimates are odds ratios (OR) with 95% confidence intervals, pooled using a random-effects model (REML method), with EVLA as the reference group; results are stratified by study design (randomized vs. non-randomized), EVLA wavelength, and follow-up time. Each square represents the OR of an individual study (square size proportional to its weight in the analysis), the horizontal line its 95% confidence interval, and each diamond the pooled subgroup or overall estimate. An OR above 1 indicates higher odds of partial occlusion with RFA relative to EVLA.

FIGURE S4 Forest plot comparing radiofrequency ablation (RFA) with endovenous laser ablation (EVLA) for reflux-free rate in patients with chronic venous insufficiency. Effect estimates are odds ratios (OR) with 95% confidence intervals, pooled using a random-effects model (REML method), with EVLA as the reference group; results are stratified by study design (randomized vs. non-randomized), EVLA wavelength, and follow-up time. Each square represents the OR of an individual study (square size proportional to its weight in the analysis), the horizontal line its 95% confidence interval, and each diamond the pooled subgroup or overall estimate. An OR above 1 indicates higher odds of a reflux-free outcome with RFA relative to EVLA.

FIGURE S5 Forest plot comparing radiofrequency ablation (RFA) with endovenous laser ablation (EVLA) for postoperative Aberdeen Varicose Vein Questionnaire (AVVQ) score in patients with chronic venous insufficiency. Effect estimates are mean differences (MD) with 95% confidence intervals, pooled using a random-effects model (REML method), with EVLA as the reference group; results are stratified by study design (randomized vs. non-randomized), EVLA wavelength, and follow-up time. Each square represents the MD of an individual study (square size proportional to its weight in the analysis), the horizontal line its 95% confidence interval, and each diamond the pooled subgroup or overall estimate. An MD below 0 indicates a lower (better) AVVQ score with RFA relative to EVLA.

FIGURE S6 Forest plot comparing radiofrequency ablation (RFA) with endovenous laser ablation (EVLA) for return-to-work time in patients with chronic venous insufficiency. Effect estimates are mean differences (MD) with 95% confidence intervals, pooled using a random-effects model (REML method), with EVLA as the reference group; results are stratified by study design (randomized vs. non-randomized), EVLA wavelength, and follow-up time. Each square represents the MD of an individual study (square size proportional to its weight in the analysis), the horizontal line its 95% confidence interval, and each diamond the pooled subgroup or overall estimate. An MD below 0 indicates an earlier return to work with RFA relative to EVLA.

FIGURE S7 Forest plot comparing radiofrequency ablation (RFA) with endovenous laser ablation (EVLA) for time to return to activities of daily living in patients with chronic venous insufficiency. Effect estimates are mean differences (MD) with 95% confidence intervals, pooled using a random-effects model (REML method), with EVLA as the reference group; results are stratified by study design (randomized vs. non-randomized), EVLA wavelength, and follow-up time. Each square represents the MD of an individual study (square size proportional to its weight in the analysis), the horizontal line its 95% confidence interval, and each diamond the pooled subgroup or overall estimate. An MD below 0 indicates an earlier return to daily activities with RFA relative to EVLA.

FIGURE S8 Forest plot comparing radiofrequency ablation (RFA) with endovenous laser ablation (EVLA) for patient satisfaction score in patients with chronic venous insufficiency. Effect estimates are mean differences (MD) with 95% confidence intervals, pooled using a random-effects model (REML method), with EVLA as the reference group; results are stratified by study design (randomized vs. non-randomized), EVLA wavelength, and follow-up time. Each square represents the MD of an individual study (square size proportional to its weight in the analysis), the horizontal line its 95% confidence interval, and each diamond the pooled subgroup or overall estimate. An MD above 0 indicates higher patient satisfaction with RFA relative to EVLA.

FIGURE S9 Forest plot comparing radiofrequency ablation (RFA) with endovenous laser ablation (EVLA) for recurrence rate in patients with chronic venous insufficiency. Effect estimates are odds ratios (OR) with 95% confidence intervals, pooled using a random-effects model (REML method), with EVLA as the reference group; results are stratified by study design (randomized vs. non-randomized), EVLA wavelength, and follow-up time. Each square represents the OR of an individual study (square size proportional to its weight in the analysis), the horizontal line its 95% confidence interval, and each diamond the pooled subgroup or overall estimate. An OR above 1 indicates higher odds of recurrence with RFA relative to EVLA.

FIGURE S10 Forest plot comparing radiofrequency ablation (RFA) with endovenous laser ablation (EVLA) for overall complication rate in patients with chronic venous insufficiency. Effect estimates are odds ratios (OR) with 95% confidence intervals, pooled using a random-effects model (REML method), with EVLA as the reference group; results are stratified by study design (randomized vs. non-randomized), EVLA wavelength, and follow-up time. Each square represents the OR of an individual study (square size proportional to its weight in the analysis), the horizontal line its 95% confidence interval, and each diamond the pooled subgroup or overall estimate. An OR above 1 indicates higher odds of complications with RFA relative to EVLA.
