# Supplementary material for: Endovenous laser ablation versus radiofrequency ablation in chronic venous insufficiency: a comparative meta-analysis of efficacy and safety
Source: Front Med (Lausanne). 2026 Aug 27;13:1816752. doi: 10.3389/fmed.2026.1816752 (PMC13558150; doi:10.3389/fmed.2026.1816752)
Supplement: Supplementary file 1 [file Table_1.DOCX]

**Table S1.** The detailed search query employed in the literature search

| No | Search Query | Result |
| --- | --- | --- |
| PubMed |  |  |
| #1 | “chronic venous insufficiency”[tiab] OR "Venous Insufficiency"[Mesh] | 9,857 |
| #2 | Endovenous[tiab] | 2,456 |
| #3 | Ablation[tiab] | 126,934 |
| #4 | Laser[tiab] OR "Laser Therapy"[Mesh] | 343,928 |
| #5 | Radiofrequency[tiab] OR "Radiofrequency Ablation"[Mesh] | 71,461 |
| #6 | #1 AND #2 AND #3 AND #4 AND #5 | 170 |
| Scopus |  |  |
| #1 | TITLE-ABS-KEY (“chronic venous insufficiency”) | 4,951 |
| #2 | TITLE-ABS-KEY (Endovenous) | 3,539 |
| #3 | TITLE-ABS-KEY (Ablation) | 258,967 |
| #4 | TITLE-ABS-KEY (Laser) | 1,644,330 |
| #5 | TITLE-ABS-KEY (Radiofrequency) | 108,861 |
| #6 | #1 AND #2 AND #3 AND #4 AND #5 | 105 |
| Web of Science | | |
| #1 | AB=“chronic venous insufficiency” | 2,313 |
| #2 | AB=Endovenous | 1,967 |
| #3 | AB=Ablation | 158,839 |
| #4 | AB=Laser | 938,380 |
| #5 | AB=Radiofrequency | 41,477 |
| #6 | #1 AND #2 AND #3 AND #4 AND #5 | 36 |
| Cochrane |  |  |
| #1 | “chronic venous insufficiency” | 673 |
| #2 | Endovenous | 713 |
| #3 | Ablation | 11,766 |
| #4 | Laser | 26,201 |
| #5 | Radiofrequency | 6,315 |
| #6 | #1 AND #2 AND #3 AND #4 AND #5 | 11 |
| Google Scholar | | |
| With all of the words | Laser ablation endovenous radiofrequency | - |
| With the exact phrase | Chronic venous insufficiency | - |
| With at least one of the words | - | - |
| Total | - | 200 |

**Table S2.** The definition criteria of major outcomes reported in included studies

| Study | Success | Complete Occlusion | Recanalization | Recurrence |
| --- | --- | --- | --- | --- |
| Almeida (2009) | Outcome not assessed | Outcome not assessed | Outcome not assessed | Outcome not assessed |
| Almeida (2006) | Outcome not assessed | Outcome not assessed | Recanalization was defined as the presence of flow in a vein segment >5 cm in length. | Outcome not assessed |
| Atik (2024) | Outcome not assessed | Definition not reported | Outcome not assessed | Outcome not assessed |
| Aurshina (2018) | Outcome not assessed | Outcome not assessed | Definition not reported | Recurrence was defined with >500ms for the GSV, SSV and ASV and >350ms for the PV. |
| Bozoglan (2016) | Outcome not assessed | Definition not reported | Definition not reported | Outcome not assessed |
| Chan (2024) | Outcome not assessed | Outcome not assessed | Outcome not assessed | Outcome not assessed |
| Dzieciuchowicz (2014) | Outcome not assessed | Outcome not assessed | Outcome not assessed | Outcome not assessed |
| El Kilic (2022) | Success was defined as the complete occlusion of the target vein and partial recanalization of a treated vein <5 mm in length. | complete occlusion of the target vein and partial recanalization of a treated vein <5 mm in length | Outcome not assessed | Outcome not assessed |
| El Mahdi (2021) | Outcome not assessed | Outcome not assessed | Outcome not assessed | Ultrasound-cnfirmed but no definition was provided |
| Eroglu (2018) | Outcome not assessed | Definition not reported | Outcome not assessed | Outcome not assessed |
| Gianesini (2021) | Outcome not assessed | Absence of flow and non-compressible GSV along 6 cm from epigastric vein confluence and distally | Definition not reported | Defined as GSV recanalization must be distinguished from the reflux reappearance. |
| Hamann (2019) | Outcome not assessed | Defined as absence of flow in a non-compressible GSV or an absent GSV at all three measurement points. | Outcome not assessed | Outcome not assessed |
| Hemsinli (2020) | Outcome not assessed | Measured by doppler ultrasonography, but definition is not provided | Outcome not assessed | Outcome not assessed |
| Irungu (2021) | Outcome not assessed | Outcome not assessed | Outcome not assessed | Outcome not assessed |
| Kempeneers (2022) | Outcome not assessed | Defined using the Modified GELEV score | Outcome not assessed | Outcome not assessed |
| Korepta (2022) | Outcome not assessed | Outcome not assessed | Outcome not assessed | Definition not reported |
| Korkmaz (2017) | Outcome not assessed | Outcome not assessed | Definition not reported | Outcome not assessed |
| Kubat (2020) | Outcome not assessed | Outcome not assessed | Outcome not assessed | Defined as new-onset varicose veins following the procedure |
| Kubat (2021) | Outcome not assessed | Complete occlusion of the vein with no reflux on the Duplex ultrasound scan in the treated GSV segment. | Definition not reported | Defined as new-onset varicose veins following the procedure |
| Lawson (2018) | Outcome not assessed | Outcome not assessed | Outcome not assessed | Outcome not assessed |
| Marston (2006) | Outcome not assessed | Complete if the saphenous vein was ablated with no flow from within 5 cm of the saphenofemoral junction (SFJ) to the knee. | Outcome not assessed | Outcome not assessed |
| Mubarak (2023) | Outcome not assessed | Outcome not assessed | Outcome not assessed | Outcome not assessed |
| Nordon (2011) | Outcome not assessed | Confirmed by duplex scanning, but no clear definition was provided. | Outcome not assessed | Defined as a 5-cm segment of GSV with reflux, identified on Duplex. |
| Park (2020) | Success was defined as the lack of flow < 3 cm throughout the treated area by duplex imaging. | Outcome not assessed | Outcome not assessed | Outcome not assessed |
| Park (2023) | Outcome not assessed | Definition not reported | Outcome not assessed | Outcome not assessed |
| Prabakar (2020) | Outcome not assessed | Outcome not assessed | Outcome not assessed | Outcome not assessed |
| Ravi (2006) | Outcome not assessed | Outcome not assessed | Outcome not assessed | Outcome not assessed |
| Ravi (2009) | Outcome not assessed | Outcome not assessed | Outcome not assessed | Outcome not assessed |
| Reem (2023) | Outcome not assessed | Definition not reported | Outcome not assessed | Definition not reported |
| Sydnor (2017) | Outcome not assessed | Outcome not assessed | Outcome not assessed | Outcome not assessed |
| Tuan (2020) | Outcome not assessed | Assessed by ultrasonography, but the definition was not provided | Outcome not assessed | Outcome not assessed |
| Yoon (2019) | Success was defined as the absence of flow/reflux on duplex ultrasound imaging on its whole length of the treated vein segment. After vein ablation treatment, patients were placed in a compressive dressing (20-30 mm Hg) with localized pressure along the treated vein and postprocedural compression stockings for several weeks | Outcome not assessed | Defined as the documentation of flow (open section >5 cm in length) in a previously treated vein | Defined as the presence of recurrent varicose veins on the treated limb that had been detected by the treating vascular surgeon |
| Ontas (2019) | Outcome not assessed | Definition not reported | Outcome not assessed | Outcome not assessed |

**Table S3.** A summary of the clinicodemographic characteristics of studies comparing RFA to EVLA in patients with chronic venous insufficiency

| **Author (YOP)** | **Operated Side (%)** | | **Bilaterality (%)** | | **Age (year)** | | | | **Gender (M/F)** | | **Varicose Vein (%)** |
| --- | --- | --- | --- | --- | --- | --- | --- | --- | --- | --- | --- |
|  | **EVLA** | **RFA** | **EVLA** | **RFA** | **EVLA** | | **RFA** | | **EVLA** | **RFA** |  |
|  | Rt/Lf | Rt/Lf |  |  | **Mean** | **SD** | **Mean** | **SD** |  |  |  |
| Almeida (2009) | - | - | - | - | 52.4 | 15.3 | 51.6 | 12.8 | 31-Oct | 29-Dec | 90 |
| Almeida (2006) | - | - | - | - | - | - | - | - | - | - | - |
| Atik (2024) | - | - | - | - | 46.24 | 11.31 | 41.56 | 10.22 | 98/314 | 74/183 | - |
| Aurshina (2018) | 736/739 | | - | - | 74.6 | 9.8 | 61.1 | 14.9 | - | - | - |
| Bozoglan (2016) | - | - | 100 | 100 | 42.8 (10.0) | | | | - | - | - |
| Chan (2024) | - | - | - | - | 55 | 13.6 | 54.4 | 13.7 | 1270/2286 | 3919/7686 | - |
| Dzieciuchowicz (2014) | - | - | 0 | 0 | 59.4 | 16.47 | 55.7 | 9.97 | 8-Jun | 7-Jun | - |
| El Kilic (2022) | 38/35 | 36/43 | 5.19 | 3.66 | 51.2 | 10.8 | 48.8 | 9.9 | 33/44 | 37/45 | - |
| El Mahdi (2021) | 7-Mar | 3-Jul | 0 | 0 | 35.6 | 6.35 | 27.2 | 6.6 | 4-Jun | 6-Apr | 100 |
| Eroglu (2018) | 70/69 | 82/67 | 0 | 0 | 45.9 | 10.4 | 44.9 | 10.5 | 66/73 | 65/84 | - |
| Gianesini (2021) | - | - | 5.26 | 7.32 | 54 | 10 | 56 | 10 | 14/24 | 16/25 | 100 |
| Hamann (2019) | 61/87 | 72/80 | 0 | 0 | 51.1 | 13.6 | 52.6 | 13.4 | 51/97 | 49/103 | - |
|  |  | 71/79 |  | 0 |  | 13.6 | 49.3 | 13.9 |  | 45/105 | - |
| Hemsinli (2020) | - | - | - | - | 46.06 | 10.15 | 43.35 | 10.08 | 42/44 | 113/158 | 100 |
| Irungu (2021) | - | - | 89 | 89 | - | - | - | - | 38/101 | | - |
| Kempeneers (2022) | - | - | - | - | 51.48 | 12883 | 51.54 | 13173 | 48/94 | 55/83 | - |
| Korepta (2022) | - | - | - | - | 63.68 | 14.51 | 63.76 | 13.92 | 46/38 | 25/21 | 26 |
| Korkmaz (2017) | - | - | - | - | - | - | - | - | 70/91 | 50/74 | 100 |
|  |  |  |  |  | - | - | - | - | 47/48 |  |  |
| Kubat (2020) | 108/146 | | 5.2 | | 44.54 | 13.62 | 45.79 | 12.16 | 23/16 | 55/66 | 14.6 |
|  |  |  |  |  | 44 | 12.97 |  |  | 21/15 |  |  |
| Kubat (2021) | - | - | - | - | 48.8 | 10.4 | 49.5 | 11.4 | 62/86 | 118/131 | 100 |
|  |  |  | - | - | 47.4 | 11.4 |  |  | 44/65 |  | - |
| Lawson (2018) | - | - | - | - | 50 | 13.75 | 49.9 | 13.57 | 43/110 | 38/120 | 100 |
| Marston (2006) | - | - | - | - | 57.6 | 14.9 | 52.5 | 11.6 | 45645 | 18/40 | - |
| Mubarak (2023) | - | - | - | - | - | - | - | - | - | - | 100 |
| Nordon (2011) | - | - | - | - | 46.7 | 14.4 | 46.9 | 15.1 | 26/54 | 34/45 | 100 |
| Park (2020) | - | - | - | - | 47.4 | 13.8 | 40.4 | 14.3 | 13/30 | 17/25 | 100 |
| Park (2023) | - | - | 6.25 | 0 | 57.2 | 14.5 | 60.3 | 12.3 | 19/29 | 20/23 | 100 |
| Prabakar (2020) | - | - | - | - | 49 | - | 47 | - | - | - | 100 |
| Ravi (2006) | 604/646 | | - | - | - | - | - | - | - | - | 91.9 |
| Ravi (2009) | - | - | - | - | - | - | - | - | - | - | - |
| Reem (2023) | - | - | - | - | 32.86 | 6.34 | 33.28 | 5.29 | Aug-57 | Oct-55 | - |
| Sydnor (2017) | 53/47 | 47/53 | 0 | 0 | 48.5 | 10.5 | 47 | 11.2 | 23/77 | 20/80 | 100 |
| Tuan (2020) | - | - | - | - | - | - | - | - | - | - | 80.4 |
| Yoon (2019) | - | - | 12.6 | 5.43 | 56.6 | 12.4 | 59.8 | 15.3 | 49/134 | 32/55 | 48.8 |
| Ontas (2019) | - | - | 0 | 0 | - | - | - | - | - | - | 100 |

YOP: Year of Publication; EVLA: Endovenous Laser Ablation; RFA: Radiofrequency Ablation; M/F: Male/Female; Rt/Lf: Right/Left; SD: Standard Deviation.
